# Tumor intrinsic and extrinsic functions of CD73 and the adenosine pathway in lung cancer

**DOI:** 10.3389/fimmu.2023.1130358

**Published:** 2023-03-24

**Authors:** Ryan R. Kowash, Esra A. Akbay

**Affiliations:** ^1^ Department of Pathology, University of Texas Southwestern Medical Center, Dallas, TX, United States; ^2^ Simmons Comprehensive Cancer Center, Dallas, TX, United States

**Keywords:** adenosine, CD73/NT5E, oleclumab, TKI - tyrosine kinase inhibitor, NSCLC, immune checkppoint blockade, metabolite

## Abstract

The adenosine pathway is an exciting new target in the field of cancer immunotherapy. CD73 is the main producer of extracellular adenosine. Non-small cell lung cancer (NSCLC) has one of the highest CD73 expression signatures among all cancer types and the presence of common oncogenic drivers of NSCLC, such as mutant epidermal growth factor receptor (EGFR) and KRAS, correlate with increased CD73 expression. Current immune checkpoint blockade (ICB) therapies only benefit a subset of patients, and it has proved challenging to understand which patients might respond even with the current understanding of predictive biomarkers. The adenosine pathway is well known to disrupt cytotoxic function of T cells, which is currently the main target of most clinical agents. Data thus far suggests that combining ICB therapies already in the clinic with adenosine pathway inhibitors provides promise for the treatment of lung cancer. However, antigen loss or lack of good antigens limits efficacy of ICB; simultaneous activation of other cytotoxic immune cells such as natural killer (NK) cells can be explored in these tumors. Clinical trials harnessing both T and NK cell activating treatments are still in their early stages with results expected in the coming years. In this review we provide an overview of new literature on the adenosine pathway and specifically CD73. CD73 is thought of mainly for its role as an immune modulator, however recent studies have demonstrated the tumor cell intrinsic properties of CD73 are potentially as important as its role in immune suppression. We also highlight the current understanding of this pathway in lung cancer, outline ongoing studies examining therapies in combination with adenosine pathway targeting, and discuss future prospects.

## 1 Introduction

The field of cancer immunotherapy has rapidly evolved over the last decade and numerous agents have received FDA approval (1). The search for new immune modulating agents is a major current focus of the broader cancer research community. Recently antibodies and small molecules targeting the adenosine pathway have gained traction as therapeutic agents for a multitude of cancer types. Numerous pre-clinical studies and clinical trials have demonstrated that the adenosine pathway is a promising therapeutic target (2–6). This is especially true in the field of lung cancer (2, 5, 7, 8). There are two key pieces of the adenosine pathway that are actively being explored as therapeutic targets including the production of adenosine itself and the receptors to which this metabolite binds (2, 3, 6). When ATP is released from the cell, a series of enzymatic events occurs on the cell surface through CD39 which converts ATP to AMP and CD73 which converts AMP to adenosine (9). There is also a non-canonical pathway leading to AMP production, but both pathways eventually converge to CD73 activity (10). CD73 is encoded by the gene *NT5E* and plays a role in numerous tumor cell intrinsic and extrinsic functions (11). Until recently, CD73 was studied and viewed mainly for its role as an ectonucleotidase involved in immune suppression, but recent studies have elucidated far more functions related to this molecule (12–14).

Adenosine is a nucleoside and is necessary for cellular functions, providing the building blocks for RNA (15). Under normal physiological conditions, extracellular adenosine levels within tissues are in the low nM concentration, however under conditions of cellular stress and cancer formation concentrations of adenosine can reach up to the 100uM range (16, 17). This has been observed within the tumor microenvironment; making targeting adenosine production a promising therapeutic strategy (15). Importantly, adenosine signaling plays a key homeostatic role throughout the body including maintaining cardiac function (18), neuronal signaling (19), and renal function (20). Regulation of adenosine levels both intracellularly and extracellularly are tightly controlled through both canonical and non canonical pathways (21). However, when there is increased expression of andenosine pathway producing enzymes, which is seen across cancer types, this equilibrium is disturbed resulting in an immune suppressive environment.

The field of lung cancer treatment has made immense strides with the addition of immune checkpoint blockade therapies to the standard of care (22–24). However, therapy resistance remains a major problem and discovering additional treatments is vital. NSCLC and a major subtype of NSCLC, lung adenocarcinoma (LUAD), has been researched most extensively in terms of the adenosine pathway (25–27). This has led to clinical trials testing adenosine pathway targeting in a subset of LUAD, EGFR mutant tumors (5, 8, 28). Additionally in NSCLC, Durvalumab, a PD-L1 antibody, is currently being tested in combination with Oleclumab, a CD73 blocking antibody, or Monalizumab, an antibody blocking inhibitory NK cell receptor NKG2A, with promising phase II results leading to the initiation of a phase III trial (2). However, in other lung cancer subtypes such as lung squamous carcinomas (LUSC), large cell neuroendocrine cancers (LCNEC), un-transdifferentiated thoracic tumors (UT), carcinoid like tumors of the lung or small cell lung cancer (SCLC) this pathway has not been thoroughly studied.

**Figure 1:**
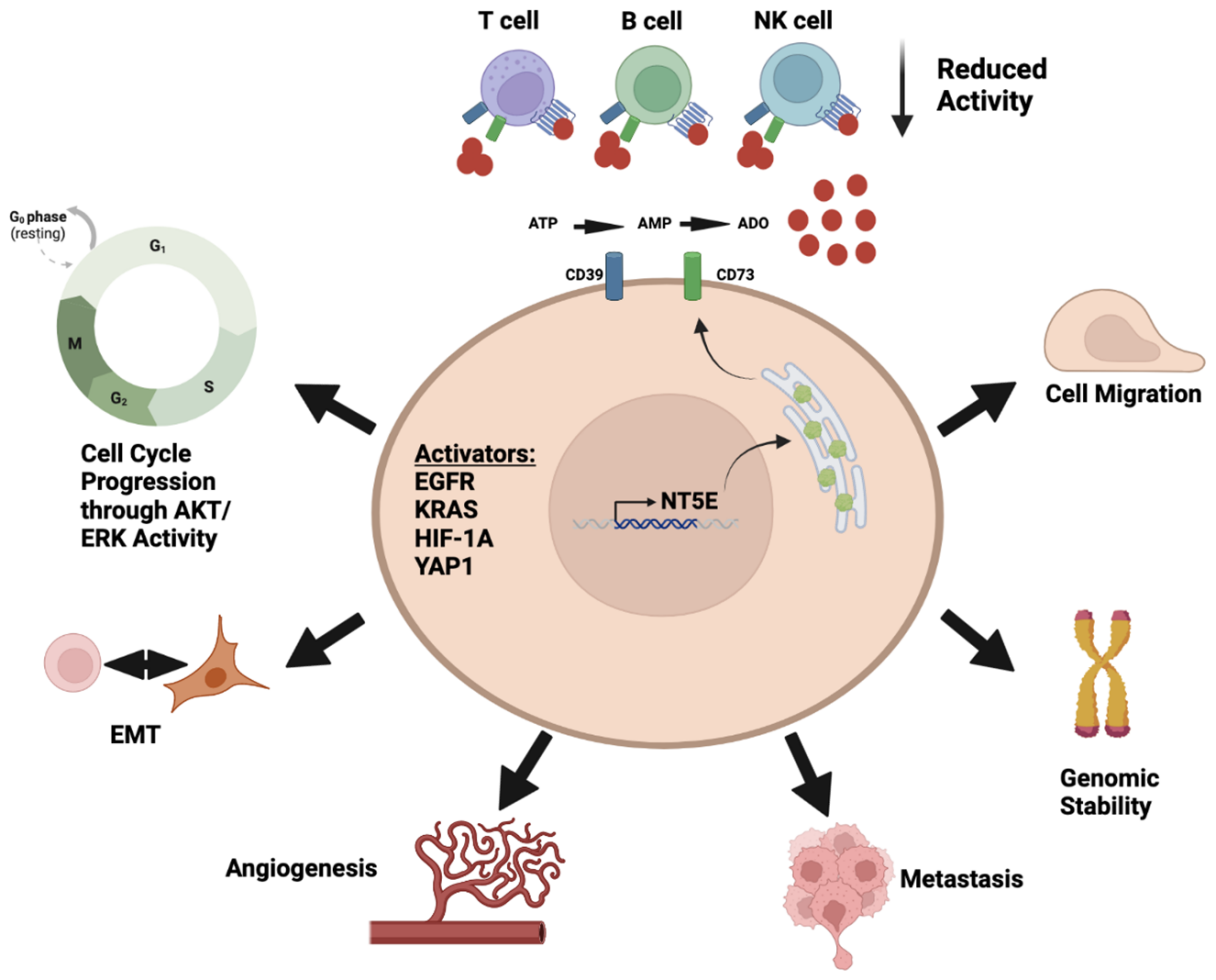
Diverse roles of adenosine pathway in the tumor microenvironment. Illustration was created using BioRender.com.

This review will outline the current status of targeting the adenosine pathway and its specific outlook in the field of lung cancer. We highlight both cell intrinsic and extrinsic properties of CD73 and the link to immune cell regulation (**Figure 1**). We provide the current understanding of this pathway in both pre-clinical models and clinical trials across lung cancer subtypes. Finally, we will outline prospects of targeting the adenosine pathway in combination with other treatment options that are already being tested in pre-clinical and clinical models.

## 2 Adenosine production pathway

There is both a canonical and non canonical pathway resulting in adenosine production. Within the canonical pathway, ATP is first released into the tumor microenvironment under various biological conditions such as hypoxia or cellular stress (29, 30). CD39, another ecto-enzyme located on the cell surface, first de-phosphorylates ATP to AMP. CD73 then dephosphorylates AMP resulting in adenosine production (31). Within the non-canoical pathway CD38 and CD203a function to convert NAD^+^ into AMP which then is converted into adenosine by CD73 (32). An additional pathway through the function of alkaline phosphatase (ALP) can convert ATP, ADP, or AMP into adenosine (33). Furthermore, prostatic acid peptidase (PAP) can convert AMP to adenosine (34). Within the cell, intracellular adenosine levels are controlled by the adenosine kinase (AK) and cyto5’NT or by S adenosyl-homocysteine-hydrolase (SAHH) (35). Intracellular adenosine is then shuttled in and out of the cell by equilibrative nucleoside transporters (36). It is important to note that the main non canonical pathway through CD38 and CD203a cannot bypass CD73 activity (32). Therefore CD73 is integral to the pathway and is responsible for most adenosine accumulation.

### 2.1 CD39 and CD73 expression in immune cell populations

It is important to note that adenosine pathway related molecules, such as CD39 and CD73, are also expressed by tumor infiltrating lymphocyte (TIL) populations (37, 38). In human colon and lung tumor samples, analysis of TIL populations found that only CD8 T cells that express CD39 represent the stimulated form of T cells whereas those lacking CD39 play a bystander role (39). An *in vitro* study found similar results that T cells rapidly upregulate CD39 expression upon activation but that CD73 expression remains unchanged (37). In contrast to these findings, another group found that CD39 positive CD8 T cells represent the exhausted phenotype with decreased expression of both TNF and IL-2 through analysis of both human and mouse samples from breast and melanoma tumors (40). It is important to note however that heterogeneity within samples was high with some TIL samples expressing high levels of CD39 and some quite low (39, 40). An additional study utilizing a melanoma mouse model found that both CD73 and CD39 are upregulated on T cells during immune response, but that high CD73 limits effector function through a decrease in mitochondrial capacity (41). This suggests a potential mechanism that T cells control levels of extracellular ATP and AMP to self regulate their activity.

In addition to T cells, CD39 and CD73 function has also been explored on NK cells. A recent report studying both breast and sarcoma tumors found that CD73 positive NK cells within the tumor express higher levels of immune checkpoint molecules such as LAG-3 compared to CD73 negative NK cells found in the peripheral blood. These NK cells have suppressive functions on CD4 T cells (42). An additional study utilizing a mouse model of melanoma found that mice with tumors show modest increase in expression of both CD39 and CD73 on NK cells as compared to tumor naïve mice (38). Furthermore, another study found that CD39 expression on NK cells was not essential for cytotoxic function (43). It is important to note that immune cell populations such as B cells (44), T regulatory cells (45), and MDSC’s (46) have also been shown to express high levels of CD39 and CD73 resulting in the production of adenosine. Interplay between the diverse cells within the tumor microenvironment resulting in adenosine production displays the complexity of this pathway.

## 3 The adenosine pathway in lung cancer

Lung cancer is the second most diagnosed form of cancer in the United States and nearly 240,000 cases were reported in 2022 (47). Around 85% of lung cancer cases in the United States represent NSCLC histology and the remaining cases are classified as SCLC (48). ICB has become standard of care in recent years due to clinical activity (reviewed in (49) and used as single agent or in combination with chemotherapy. ICB was also explored in the neo-adjuvant setting in early stage NSCLC and due to clinical benefit received FDA approval in combination with chemotherapy in 2022 (50). In contrast, combination of PD-L1 blockade and chemotherapy, while receiving FDA approval, has shown limited clinical benefit in SCLC (51). However, for a subtype of SCLC, known as non-neuroendocrine characterized by a lack of expression of neuroendocrine genes, there has been some benefit with the addition of ICB therapy (52). Treatment for LCNEC and other lung cancers are typically chemotherapy as well (53, 54). The role of ICB in this lung cancer subtype is not yet well studied but there has been some response seen in small clinical trials (55). There is a need to look more extensively at immunosuppressive pathways, such as the adenosine to overcome therapy resistance and tailor treatment options to the individual patient (56–58).

Among all lung cancer subtypes, CD73 has been mostly studied in LUAD. Compared to normal lung, LUAD has significantly increased CD73 expression demonstrating that high CD73 is a tumor specific characteristic (27, 59). CD73 has been also detected in LUSC in some cases but is not universal (59, 60). One study of CD73 in LUSC found that similar to LUAD that knockdown of CD73 results in decreased cell proliferation, migration, and increases response to TKIs (61). In SCLC, adenosine pathway expression was reported in some patient samples and circulating tumor cell explants (CDX) models (62, 63).

Multiple studies have demonstrated across cancer types that expression of genes coding for molecules in adenosine pathway is correlated with poor survival and low overall response rate to cancer therapies (64–66). Recently, adenosine pathway expression in LUAD and LUSC has been investigated and similar results were seen with high CD73 expression on tumor cells correlating with disease progression, treatment relapse, and poor survival (60). Interestingly, in this same study they found that high adenosine receptor expression was associated with increased survival and that correspondingly high CD73 and low adenosine receptor expression resulted in the worst survival outcomes (60).

### 3.1 Adenosine pathway expression in EGFR mutant LUAD

Recently, multiple groups have focused on targeting the adenosine pathway in EGFR mutant NSCLC leading to swift discoveries in the recent years (26, 67). Standard of care for EGFR mutant tumors are tyrosine kinase inhibitor (TKI)s (68). EGFR mutant tumors are not very responsive to ICB therapy possibly due to their low tumor mutational burden (TMB) as compared to tumors carrying another common oncogenic driver of NSCLC, KRAS, which are more responsive to ICB (69, 70). Somatic mutations can serve as neoantigens and high TMB status is associated with increased sensiviity to ICB therapies on NSCLC (71). Response rates to ICB therapies are between 7-16% depending on EGFR mutation type, compared to 22% in EGFR wild type tumors (72). Acquired resistance to TKIs, such as Osimertinib is common and occurs on average around 18 months after treatment initiation (73). Multiple studies reported that CD73 upregulation was one of the mechanisms of resistance to TKIs (74, 75). We recently analyzed genes related to immune cell function in EGFR mutant versus wild type LUAD and found that *NT5E* and *ADORA1*, which encodes for one of the adenosine receptors, were two of the top upregulated immune-suppresive genes in this subtype (26). EGFR mutant NSCLC has a suppressed tumor microenvironment with few NK cells and CD8 T cells, but increased dendritic cell populations, and other immune cell populations remain similar to those seen in EGFR wild type tumors (26). In an EGFR mutant mouse model, we showed that treatment with a CD73 antibody alone resulted in decreased lung tumor growth displaying proof of concept as a treatment option (26). Another group observed similar correlations with EGFR pathway activation and adenosine signaling. Interestingly this group showed Oleclumab is effective in treating EGFR mutant tumors in combination with PD-L1 antibody in a human peripheral blood mononuclear cell (PBMC) transplanted mouse model (76).

Another recent report focused on the connection between *MET* amplification, CD73 and suppression of STING pathway in EGFR mutant tumors (67). Authors found that *MET* amplification induces CD73 expression and restrains the function of STING resulting in reduced T cell activation while also causing resistance to 3^rd^ generation EGFR TKIs. MET amplification was found to be specifically associated with CD73 expression in EGFR mutant LUAD. Genetic knockout of MET in lung cancer models resulted in decreased CD73 expression therefore displaying a connection between these two pathways (67). This study links CD73 intrinsic function to extrinsic function through immune cell regulation.

### 3.2 Adenosine pathway expression in other common oncogenic drivers of LUAD

Although adenosine pathway expression has been studied the most in the EFGR mutant subset of LUAD, other common mutations such as KRAS, BRAF, and MET are just beginning to be explored. A recent study utilizing the TCGA data set found that CD73 expression is significantly elevated in KRAS mutant LUAD (25, 77). Similar induction of CD73 expression was also seen with *ALK* gene alterations. As proof of concept, they found that pharmacological inhibition of ALK led to a decrease in CD73 mRNA expression (25). This data suggests a link between the adenosine pathway and oncogenic drivers of LUAD. A new study in pancreatic ductal adenocarcinoma (PDAC), where almost 80% of cases are KRAS mutant, demonstrated that activating mutation in KRAS alone is sufficient to elevate levels of CD73 suggesting that CD73 induction with oncogenes extends beyond lung cancer (78). Results from these studies thus far demonstrate oncogenic drivers of NSCLC possibly drive increased CD73 expression and that underling mechanism behind this warrants further investigation.

### 3.3 Adenosine pathway expression in SCLC

CD73 expression is seen in a subset of SCLC patient CDX models and cell lines and its expression correlated with the non-neuroendocrine or YAP1 positive subtype (62, 63). A recent study focused on the metabolomics of primary SCLC tumors. This study demonstrated that AMP and adenosine were the two significantly upregulated metabolites in primary patient samples in the MYC high subtype versus MYC low subtype (79). This study also examined the link between chemoresistance and purine biosynthesis and found that chemo-resistant SCLC cell lines have increased levels of AMP and ATP compared to parental cell lines. These pre-cursors could result in adenosine accumulation within the tumor microenvironment (79). A similar finding was seen *in vivo* with chemotherapy relapsed tumors displaying increased purine biosynthesis, including AMP (79). Therefore these interesting findings suggest that purine biosynthesis maybe advantageous for SCLC relapse after treatment.

### 3.4 Adenosine pathway inhibition in the context of lung cancer tumor transdifferentiation and heterogeneity

Cellular plasticity and evolution of the tumor microenvironment with disease progression has been reported in NSCLCs. Transdifferentiation of EGFR mutant NSCLC to SCLC is one of the reported resistance mechanisms to EGFR TKIs. This occurs in approximately 3-10 percent of EGFR mutated NSCLCs (80). Studies have found that SCLC transformed tumors in these patients no longer respond to TKIs but they do respond to platinum etoposide therapy at least initially, similar to SCLCs (80–82). A recent report found that loss of extracellular-signal regulated kinase (ERK) signaling dependency was the main regulator in this transformation and this resulted in expression of neuroendocrine transcription factors which was normally repressed by ERK signaling (83). SCLC transdifferentiation was also reported in therapy resistant KRAS mutant LUAd (84). It would be interesting to study whether these transformed tumors retain expression of CD73 and can be targeted by CD73 targeting molecules.

In the field of SCLC multiple groups have found that SCLC primarily switches from neuroendocrine to a more non-neuroendocrine like state. This was shown to be driven through NOTCH driven Myc expression (85, 86), while other groups show that this is driven by EMT gene signature including expression of YAP1 (87). Neuroendocrine and non-neuroendocrine tumor types have shown to have different immune microenvironments with neuroendocrine tumors having far less immune cell infilrates (48, 88, 89). A new report examined 146 SCLC patient samples through IHC and found that 2.3% of tumors expressing YAP1 dominantly. Additionally, the authors found that 17.6 percent of tumors expressed 2 of the SCLC lineage markers and 2.8% were positive for 3 different lineage markers. These areas of different lineage marker staining tended to cluster away from one another suggesting that different cell populations were in various parts of the tumor (90). A recent study highlights that the SCLC intertumoral composition is constantly evolving and that there maybe continuous subtype switching (91). Multiple studies have suggested that one of the target genes of the YAP/TAZ axis is CD73, suggesting the idea that induction of CD73 expression may provide a selective advantage to YAP1 expressing SCLC cells under treatment (92–94). This connection must be explored further, but it is possible that the YAP1 positive cells are contributing to adenosine production, with high expression of CD73 seen in YAP1 positive CDX and SCLC cell line models (63, 95).

## 4 Tumor cell intrinsic functions of CD73

CD73 has been demonstrated to be involved in numerous cancer processes including metastasis (12), increased cell proliferation (96), and tumor invasion (97). In addition to its membrane bound form, CD73 is expressed in soluble forms (98), on extracellular vesicles (99), and localized within different cellular compartments of tumor cells (100). Studies on the intrinsic properties of CD73 have been investigated in numerous cancer types including liver cancer (101), GI cancer (102), Glioblastoma (103), and lung cancer (104) (**Table 1**). CD73 has been implicated as a target of transcription factors including YAP1 (92), SNAIL (105), HIF1A (106), and TGFB (14, 107, 108). Transcriptional regulation by key drivers of cancer suggests that an increase in CD73 expression is advantageous for cancer cell growth. Therefore, understanding the mechanism of CD73 intracellular function is key to understanding the overall biology of this molecule.

**Table 1:** Recent studies highlighting tumor cell intrinsic functions of CD73.

Tumor type	Subtype	Model Used	Conclusions	Reference
**Lung**	*EGFR* mutant LUAD	Human NSCLC lines HCC827 and PC9	Modulates response to TKI therapy through EGFR-ERK signaling	(77)
**Lung**	EGFR, KRAS, or ALK mutant LUAD	Panel of human NSCLC cell lines with EGFR, KRAS, or ALK mutations	Expression is modulated by hypoxia and glucose deprivation and is higher in metastatic lessions possibly as a resistance mechanism	(25)
**Lung**	EGFR and *MET* mutant *LUAD*	Multiple human NSCLC cell lines and patients samples with *EGFR mutations*	Restrains STING immuogenecity in *MET* driven LUAD	(67)
**Breast**	TNBC	Human MDA-MB-231 and mouse 4T1 TNBC cells	Promotes the EMT program and cell invasion	(12)
**Breast**	Mesenchymal vs. Epethelial Breast Cancer models	PyMT breast cancer model of *Snail* high and *Snail* low cells modeling mesenchymal vs. epethelial breast tumors	Plays a role in the EMT program	(111)
**Glioma**	Glioblastoma	GL261 mouse glioma model and U251 human glioblastoma cells	Regulates angiogenic factors that lead to tumor growth and metastasis	(116)
**GI Cancer**	Gastric Cancer	Human Gastric Cancer cell lines MKN45, SGC7901, AGS, MGC803, and BGC823 and GI mucosal cell line GES-1	Promotes cancer cell migration and modulates the cytoskeletal regulation pathway	(102)
**GI Cancer**	Colorectal Cancer	Colorectal cancer cell lines RKO, SW480, HCT-15, LoVo and KM12	Plays a role in cell cycle progression and cell growth	(112)
**Pancreatic Cancer**	Pancreatic Ductal Adenocarcnoma	Human PDAC cell lines PANC-1, AsPC-1, BxPC-3, L3.7, MIA PaCa-2, and SW1990 as well as paired normal and tumor patient samples	Contributes to gemcitabine resistance throuhg *AKT* signaling	(100)
**Pancreatic Cancer**	Pancreatic Ductal Adenocarcnoma	Human PDAC cell lines PANC-1, AsPC-1, BxPC-3, L3.7, MIA PaCa-2, and SW1990 as well as paired normal and tumor PDAC patient samples	Plays a role in cell cycle progression and apoptosis	(114)
**Liver cancer**	Heptatocellular Carcinoma	Primary HCC samples and HCC cell lines HCCLM3, Hep3B, MHCC97L, and HepG2	Regulates sphere-forming capacity and promotes cell stemness	(13)
**Thyroid Cancer**	Papilarry Thyroid Carcinoma	PTC cell lines K-1 and SNU-790 as well as PTC primary patient samples	Plays a role in cell migration, cell cycle arrest, and EMT	(124)
**Endometrial Cancer**	Endometrial Adenocarcinoma	Endometrial cancer cell lines HEC-1-A and HEC-50, endometrioid endometrial carcinomas	Interacts with TGF-β1 function	(14)

### 4.1 Role of the CD73 in the EMT program

Recent studies have shown that CD73 contributes to EMT (epithelial mesenchymal transition) and is a novel target beyond the current key genes that are implicated in EMT such as E-cadherin, vimentin, and N-cadherin (109). A recent study utilizing a breast cancer model with genetic loss or pharmacological inhibition of CD73 resulted in far fewer and less invasive organoids *in vitro* and decreased lung metastasis when injected into an immune competent mouse model. It was reported that when CD73 is knocked down that there is increased E-cadherin expression and that cells appeared to revert from EMT (12). A similar finding was also shown in a preclinical model of ovarian cancer (110). Another recent study in breast cancer found that SNAIL, a key regulator of EMT, bound at promotors of *NT5E* through chromatin immunoprecipitation (ChIP) sequencing analysis suggesting that there could be interplay between these two genes and the EMT program (111). An additional recent report in NSCLC line A549 showed that CD73 overexpression leads to increased invasion and metastasis both *in vitro* and *in vivo* (25). Further studies employing the A549 model demonstrated that CD73 promotes cell proliferation through binding to EGFR which leads to activation of AKT/mTOR pathway (104). Across numerous cancer types genetic knockdown or knockout of CD73 in human cancer cell lines grown in immune deficient mice demonstrated that loss of this molecule reduces tumor growth and metastatic potential even when a functional immune system is not present (12, 110, 112, 113).

### 4.2 The role of CD73 in cell cycle regulation and treatment resistance

Outside of the scope of the EMT program, studies have demonstrated that CD73 plays a role in other biological processes as well. A few different studies have linked CD73 to control of cell cycle progression (112, 114, 115). A specific study in pancreatic ductal adenocarcinoma (PDAC) found that when CD73 is knocked down, this results in arrest of cells at G1 phase through AKT/ERK/Cyclin D signaling (114). Other studies have focused on how CD73 expression leads to treatment resistance. A recent *in vitro* study in LUAD demonstrated that knock down of CD73 altered cell cycle progression and sensitized cells to cisplatin chemotherapy treatment (115). Similar results were seen in a model of glioblastoma when they knock down CD73 in their model this resulted in increased temozolomide sensitivity, and that resistance is caused by CD73-A_2B_AR signaling (116). An additional *in vitro* study in breast cancer model found that CD73 deficient MDA-MB-231 cells to be significantly more sensitive to Olaparib likely due to decreased PARP activity. They also found that loss of CD73 suppressed mitochondrial respiration and led to increased genomic instability (117).

Studies thus far have demonstrated an interesting link between expression of CD73 and sensitivity to commonly used therapeutic agents. Of note, in the tumors resistant to numerous therapies CD73 expression is increased. These include radiation (118), chemotherapy (119), TKIs (75), as well as monoclonal antibodies (120). This has been shown to occur through different mechanisms across cancer types. A study in melanoma found that there was increased CD73 expression in the tumors resistant to both adoptive T cell transfer and ICB therapy. They found that both in mouse models and human tissues that MAPK signaling resulted in class switching from an epethial to mesenchymal state and that there was clonal selection for a more “invasive” cell population (121). In hepatocellular carcinoma (HCC) CD73 was shown to contribute to resistance to Lenvatinib, a VEGF inhibitor, through AKT overactivity which resulted in increased SOX9 expression and stemness of HCC cells (13). Within the tumor micronevironment, a pre-clinical study in triple negative breast cancer (TNBC) found that after chemotherapy that there was an increase in CD73, CD47, and PD-L1 positive tumor cells (122). Such examples of therapy resistance show that CD73 is tied to multiple mechanisms of therapy resistance.

### 4.3 Non- enzymatic functions of CD73

CD73 expression correlated with adenosine production in most studies, however it is technically difficult to separate the ectonucleotidase activity from other functions of CD73 in genetic inactivation or antibody blockade studies. One group recently thoroughly studied the non-enyzmatic role of CD73 by blocking the nucleotidase function. They reported a direct physical interaction between CD73 and Src in the ER resulting in Src activation (100). They found that CD73 ectonucleotidase activity did not contribute to Gemcitabine resistance but that resistance was rather caused by AKT pathway activation by CD73. Furthermore they demonstrated that when they mutated the zinc finger binding domain of CD73, which severely inhibits ectonucleotidase activity, that there was still resistance to Gemcitabine suggesting again that it was not the enzymatic activity leading to resistance (100). An additional study in HCC showed that CD73 localizes within the ER of cancer cells and is more abundant in tumors than normal tissue. They propose that there is both a short and and full length of CD73 proteins with the long form only having ectonuclotidase activity and the shorter version found solely localized in the ER (123). To our knowledge these are the only two studies to yet examine proximity of CD73 in the cell and therefore further understanding of the differences between mebrane bound and intracellular CD73 are needed.

## 5 Tumor cell extrinsic functions of adenosine in the tumor microenvironment

Once adenosine is released into the tumor microenvironment it plays both an immunosuppressive role for some immune subtypes and activates others, although there is less evidence for the latter. There are a total of four adenosine receptors found on the cell surface including A_1,_ A_2_A, A_2_B, and A_3_ (21). These receptors are expressed on cell types throughout the body including both innate and adaptive immune cells. The A_2_AR receptor is expressed on T, NK, and B cells and when bound to adenosine this results in loss of activity in cell types (125–127). The A_2_BR receptor is expressed on macrophages, monocyte derived suppressor cells (MDScs) and cancer associated fibroblasts (CAFs) and upon binding of adenosine results in their activation leading to immune suppresion (128). Another important immune cell type that expresses the A_2_BR are dendritic cells (DC), which are key to antigen presentation (129). The literature demonstrates mixed findings thus far into this cell type with studies showing that adenosine impairs DC migration but does not affect their function (130). In contrast, another study found adenosine impairs DC function and that these impaired DCs release angiogenic cytokines promoting tumor growth (131). Numerous studies have demonstrated that increased adenosine production results in decreased activity of cytotoxic T cell populations and increased activity of regulatory T cell (Treg) populations (125, 132, 133). This combined signaling across immune cell types leads to an immunosuppressive tumor microenvironment.

### 5.1 Role of adenosine pathway on NK cell function

NK cells are a key component of the innate immune system and display cytotoxic reponse. Recent advances in the field of lung cancer have shown that NK cells play an essential role in controlling tumor growth and response to ICB therapy (134–137). NK cells can target cancer cells independent of antigen presentation on major histocompatibility complex (MHC) making them a valuable therapeutic target as MHC-I expression is lost or reduced in tumors as one of the mechanism of immune evasion (138). Loss of MHC-I is seen across lung cancer subtypes as well (139). However, loss of MHC-I can sensitize tumors to NK cell killing as MHC-I is an immune inhibitory molecule for NK cells (140). Recent advances in the field of lung cancer have shown that NK cells play an essential role in controlling tumor growth and response to ICB therapy (134–137). Recent studies have demonstrated that around 10-20 percent of circulating lymphocytes in the lungs are NK cells, which is higher than NK cell levels found in peripheral blood (141). However, there are numerous reasons why these cells are not able to infiltrate tumors, and reasons include different immune suppressive metabolites and inhibitory molecules. Additionally, tumors have been shown to shed the NK cell activating ligands to escape NK cell tumor killing and antibodies are currently being tested that can prevent this (142).

It was reported that adenosine impedes NK cell function (143–145). A_2_AR inhibition in a co-culture system resulted in increased NK cell proliferation and signaling response as compared to untreated cultures (146). As a mechanism of tumor resistance researchers found that upon binding of the 4-1BBL domain that tumor cells can hijack NK cells and induce CD73 expression in the NK cells in a breast cancer model. These adenosine producing NK cells additionally had higher levels of immune inhibitory checkpoints such as LAG-3 (42). A similar finding was also found in *in vitro* co culture methods when cancer cell lines with high CD73 were found to induce CD73 expression in an established NK cell line, NK-92 cells. Cancer cell lines with low CD73 expression did not induce CD73 expression in NK cells (147). A recent study used a chimeric antigen receptor (CAR)-NK cells to target CD73 in a preclinical lung cancer model. Authors observed that both *in vitro* and *in vivo* that their CD73 directed CAR inhibited lung cancer growth while not attacking normal tissue (126). These findings demonstrate that adenosine as a cancer target must be explored further.

## 6 Clinical targeting of adenosine pathway in lung cancer

The first clinical stage antibody developed to target the adenosine pathway was created by Medimmune, compound MEDI9447 (Oleclumab), and began clinical trial testing in 2015 (148). Since then, many studies were initiated to target various components of this pathway including CD73 and CD39 followed by A_2_AR and combined A_2_AR/A_2_BR antibodies (149).

Trials for CD73 targeting agents have progressed the most reaching phase III testing (150). Trials are ongoing for NSCLC (2), PDAC (151), and other advanced solid tumors. A recent phase Ib/2 trial testing Oleclumab in combination with Osimertinib in EGFR mutant NSCLC demonstrated safety and efficacy and is now proceeding to phase II trials (3). The most exciting trial thus far testing Durvalumab alone or in combination with Oleclumab or Monalizumab in stage III unresectable NSCLC recently progressed to phase III testing. At median follow up of 11.5 months the ORR in Durvalumab plus Oleclumab was 30%, and Durvalumab combined with Monalizumab ORR was 35.5%. PFS was also higher in the combination groups as compared to Durvalumab alone (2).

CD39 has also been explored as a therapeutic target. In pre-clinical models, molecules targeting CD39 have shown promising results in colon cancer (152), melanoma (153), and ovarian cancer (154). CD39 antibodies are currently being tested in combination with ICB therapy (NCT04336098) and chemotherapy (NCT03884556) and are currently in phase I stage (155).

Targeting the adenosine receptor has shown modest effect and the trial in combination with Oleclumab in EGFR mutant NSCLC was stopped due to safety issues and lack of efficacy (150). However, in advanced prostate cancer there was some response with single agent AZD4635 (A_2_AR antagonist) with an overall response rate of 5.1% and when combined with Durvalumab combination, response rate was 16.1% (5).

## 7 Discussion

The study of the adenosine pathway as a therapeutic target is still in its early stages, however preclinical studies and clinical trial data have demonstrated that targeting this pathway is a viable therapeutic strategy moving forward. Increasing evidence has demonstrated that CD73 has roles independent of its enzymatic function. However only one group thus far has thoroughly studied this mechanism. Even with their findings however it is still thought provoking whether the intracellular function of CD73 is the same as the canonical econucleotidase activity on the cell membrane. This raises an important question as current antibodies targeting CD73 would therefore not prevent activity within the intracellular space. We propose that based on the literature generated thus far that CD73 intracellular activity, whether enzymatic or not, could possibly play an important role in cancer progression as the immune suppression aspect through adenosine production. We note that although we have focused this review around CD73 and adenosine, we highlight that both CD39 as well as the adenosine receptors are also important pieces of this pathway. Importantly studies are needed to understand whether CD39 or CD73 blockade leads to increased compensatory activity of non-canonical pathways adenosine pathways such as CD38 and CD203a.

Targeting the adenosine pathway with recently initiated clinical trials has grown as new combinatory approaches are tested in lung cancer. We believe that CD73 inhbition as a therapeutic target can be applied to other lung cancer types outside of the current scope of EGFR mutant LUAD and unresectable NSCLC. This is especially important surrounding the topic of lineage plasticity and acquired treatment resistance. Therefore we need to better understand as a field what is driving this plasticy and how this is leads to a lack of response to therapeutic agents. We believe an important aspect of further study is understanding how EGFR NSCLCs transdifferentiate to SCLC and whether blockade of the adenosine pathway could be a potential treatment for these tumors.

We also believe there is an unmet need to better understand the role of NK cells and activating this cytotoxic cell type as they have been shown to play critical roles in both NSCLC and SCLC. In regards to the clinical trial data with the ICB and Oleclumab or ICB and Monalizumab COAST study, we suggest a potential further clinical trial could test Monalizumab plus Oleclumab. We believe that based on the current pre-clinical and clinical data thus far that it does not appear that targeting the adenosine pathway alone will result in drastic therapeutic benefit and therefore the best combinatory therapeutics and dosing schedules warrant further investigation. To our knowledge adenosine pathway expression in LCNEC and other thoracic tumors of the lung have not been explored extensively. Several groups have reported that LCNEC and undifferentiated tumors of the lung are becoming more common, and they have not been studied well, but interestingly these tumor types likely arise from therapy resistance (156). Concluding, we see many avenues of further study that are needed both in the pre-clinical and clinical space in order to further understand the role of CD73 and the adenosine pathway overall across not just lung cancer but, also other cancers with an activated adenosine pathway.

## Author contributions

Concept and design: RRK and EAA. All authors contributed to the article and approved the submitted version.
